# *Matters arising*: personalised viscoelastometry-guided systemic thrombolysis for high- and intermediate-high-risk acute pulmonary embolism in the ICU: a single-centre randomised controlled interventional feasibility trial

**DOI:** 10.1186/s40635-026-00913-5

**Published:** 2026-06-15

**Authors:** Maajid Mohi Ud Din Malik, Rajesh Prasad Jayaswal

**Affiliations:** 1 Dr. D.Y. Patil School of Allied Health Sciences, Dr. D.Y. Patil Vidyapeeth (Deemed to Be University), Sant Tukaram Nagar, Pimpri, Pune, 411018 India; 2 University Institute of Allied Health Sciences, Chandigarh University, Mohali, Punjab, 140413 India

We read with interest the single-centre randomised feasibility trial by Kállai and colleagues, which evaluated a viscoelastometry-guided, low-dose, prolonged systemic thrombolysis protocol for high- and intermediate-high-risk acute pulmonary embolism [1]. Conducting a randomised trial of thrombolysis in critically ill patients with acute pulmonary embolism is logistically and ethically demanding, and the investigators should be commended for completing recruitment in this fragile cohort and for an explicit feasibility design. The clinical question is timely, and the proposed individualisation strategy is conceptually appealing. We raise a number of methodological observations that, in our view, bear on how the reported safety and efficacy signals are best interpreted, and that may be useful for the design of subsequent confirmatory work.

## Post-randomisation exclusions and the intention-to-treat principle

A total of 22 patients were randomised, 8 to the control group (CG) and 14 to the viscoelastometry-guided group (VGG), with 7 and 12 patients, respectively, included in the final analysis. The CG participant in whom thrombolysis was discontinued because of severe chest pain is shown in Fig. 2 as having been allocated but not retained for the primary analysis (*n* decreasing from 8 to 7); a brief justification, ideally with a parallel intention-to-treat sensitivity analysis, would help readers gauge the impact of this exclusion, since chest pain during thrombolysis may itself constitute a treatment-related event. We further note that two VGG participants (one with subsequently diagnosed pulmonary angiosarcoma and one with pre-existing pulmonary hypertension) were excluded post hoc, although Fig. 2 depicts only the angiosarcoma exclusion (with the analysed VGG denominator displayed as *n* = 13), whereas the main text reports the analysed VGG denominator as *n* = 12 with both exclusions described. Reconciling the figure and the text would aid CONSORT-compliant reporting [2]. We recognise that the authors explicitly frame the trial as feasibility-oriented and not designed for hypothesis testing; nevertheless, transparent handling of post-randomisation exclusions remains relevant for any subsequent confirmatory analysis.

## Reporting of randomisation procedures

The authors transparently disclose that no allocation ratio was prespecified and that group-size imbalance was considered acceptable in this feasibility context (original article, Methods). We accept this framing. We would, however, suggest that the manuscript additionally specify the sequence-generation method (block sizes, software used), the procedure for allocation concealment, and the personnel responsible for sequence generation and assignment. These details are recommended by the CONSORT 2010 pilot and feasibility extension [3] and would strengthen the methodological transparency of the report without altering its feasibility framing.

## Outcome assessment for right ventricular recovery

Resolution of right ventricular (RV) dysfunction was operationalised as disappearance of the D-shaped left ventricle on transthoracic echocardiography, performed by a single investigator (A.K.) who was also the lead author and was unblinded to allocation. Given that this is the principal efficacy outcome––reported as 100% resolution in the VGG versus 66% in the CG––the design is susceptible to detection bias, a limitation the authors themselves acknowledge in their Limitations section. ESC guidance recognises several complementary echocardiographic markers of RV pressure overload, including TAPSE, the RV/LV ratio, pulmonary acceleration time, and the eccentricity index [4]. We acknowledge that blinded core-lab adjudication and multi-observer reading are not always feasible in a single-centre pilot study; nonetheless, future iterations of this work would benefit from a composite definition assessed by a blinded reader, which would also facilitate pooled analyses across centres.

## Adjudication of major bleeding events

The fatal bleeding event in the CG, occurring after cardiopulmonary resuscitation, is reported as therapy-related, whereas the intracranial haemorrhage in the VGG is described by the authors as “possibly linked to an unreported prior collapse may have contributed to the outcome.” The qualifying language itself signals the difficulty of definitive attribution. Under ISTH criteria, both events would conventionally be classified as major bleeding regardless of contributory mechanism [5], with attribution recorded separately as a secondary descriptor. Uniform adjudication, ideally by an independent committee, would reduce the risk of differential ascertainment between groups. We recognise that with three major bleeding events across 19 analysed patients the data cannot, in any case, support firm comparative safety inferences, and we suggest the safety section be framed accordingly.

## Derivation of the lysis-time threshold

The proposed lysis-time threshold of > 2020s was identified by maximising the Youden index in the same dataset used to evaluate its discriminative performance. This in-sample optimisation procedure is well recognised to overestimate true classification accuracy, and the derived clinical zones (< 1800s “danger,” > 2400 s “green”) inherit that optimism. The authors themselves describe these analyses as exploratory and state that the thresholds “need to be validated in different clinical contexts;” we strongly endorse that caveat and would suggest that it be repeated explicitly in the Conclusions, since it is the discussion that readers and downstream clinical adopters are most likely to encounter. Prospective external validation in an independent cohort is required before these thresholds are applied at the bedside.

## Alignment between feasibility design and the framing of conclusions

The trial is described as a feasibility study and was not powered for efficacy comparisons, as the authors explicitly acknowledge. We note that the discussion does already include appropriate hedging (“cautiously suggest,” “hypothesis-generating,” “no definitive conclusions can be drawn”). However, the abstract and several headline statements within the Results-significantly lower rtPA dose, full RV recovery in the VGG, and sustained AP-test maximum clot firmness––read as comparative safety and efficacy claims that, taken alone, may be carried forward in citation without their hedging. With seven control patients, between-group differences of any magnitude are accompanied by wide confidence intervals, and established frameworks for pilot and feasibility trials caution against drawing directional inferences from such data [3]. We suggest, in the spirit of the authors’ own design statement, that the abstract and Results headlines be aligned with the more cautious framing already present in the Discussion.

In summary, the protocol is intellectually attractive and the dataset is genuinely informative for hypothesis generation; we recognise the difficulty of conducting any randomised trial in this patient population. The combination of post-randomisation exclusions with a figure–text discrepancy, single-operator unblinded outcome assessment, asymmetric attribution of bleeding events, and in-sample threshold derivation does, however, limit the strength of the comparative safety and efficacy inferences advanced in the Results and abstract. A multicentre randomised trial incorporating prespecified intention-to-treat analysis, blinded core-lab echocardiographic adjudication, harmonised bleeding adjudication by an independent committee, and prospective external validation of the proposed lysis-time thresholds would, we believe, build constructively on the present work and address each of these points.

## Data Availability

Not applicable. This correspondence reports no primary data.

## Abbreviations

CG: Control group
CONSORT: Consolidated Standards of Reporting Trials
ESC: European Society of Cardiology
ICU: Intensive care unit
ISTH: International Society on Thrombosis and Haemostasis
ITT: Intention-to-treat
LT: Lysis time
MCF: Maximum clot firmness
rtPA: Recombinant tissue plasminogen activator
RV: Right ventricle
RV/LV: Right ventricle-to-left ventricle ratio
TAPSE: Tricuspid annular plane systolic excursion
VGG: Viscoelastometry-guided group
